# An Empirical Investigation of Dance Addiction

**DOI:** 10.1371/journal.pone.0125988

**Published:** 2015-05-07

**Authors:** Aniko Maraz, Róbert Urbán, Mark Damian Griffiths, Zsolt Demetrovics

**Affiliations:** 1 Department of Clinical Psychology and Addiction, Eötvös Loránd University, Budapest, Hungary; 2 Doctoral School of Psychology, Eötvös Loránd University, Budapest, Hungary; 3 Department of Personality and Health Psychology, Eötvös Loránd University, Budapest, Hungary; 4 Nottingham Trent University, Nottingham, United Kingdom; University of Ariel, ISRAEL

## Abstract

Although recreational dancing is associated with increased physical and psychological well-being, little is known about the harmful effects of excessive dancing. The aim of the present study was to explore the psychopathological factors associated with dance addiction. The sample comprised 447 salsa and ballroom dancers (68% female, mean age: 32.8 years) who danced recreationally at least once a week. The Exercise Addiction Inventory (Terry, Szabo, & Griffiths, 2004) was adapted for dance (Dance Addiction Inventory, DAI). Motivation, general mental health (BSI-GSI, and Mental Health Continuum), borderline personality disorder, eating disorder symptoms, and dance motives were also assessed. Five latent classes were explored based on addiction symptoms with 11% of participants belonging to the most problematic class. DAI was positively associated with psychiatric distress, borderline personality and eating disorder symptoms. Hierarchical linear regression model indicated that Intensity (*ß*=0.22), borderline (*ß*=0.08), eating disorder (*ß*=0.11) symptoms, as well as Escapism (*ß*=0.47) and Mood Enhancement (*ß*=0.15) (as motivational factors) together explained 42% of DAI scores. Dance addiction as assessed with the Dance Addiction Inventory is associated with indicators of mild psychopathology and therefore warrants further research.

## Introduction

Dancing has substantial benefits for physical and mental health such as decreased depression, anxiety and increased physical and psychological wellbeing [1–4]. At the same time, little is known about the psychological underpinnings of excessive dancing. Although there are theoretical reasons to assume that excessive dancing may have harmful effects for the individual, there have been very few studies that have studied the effects of excessive dance activity, and none that have described the psychological factors and consequences associated with dance addiction.

Given the lack of empirical research in dance addiction, exercise addiction is arguably similar and could be considered as conceptually resembling dance addiction for several reasons. For example, Pierce et al. [5] found that dancers score higher on the Exercise Addiction Scale compared to endurance and non-endurance athletes. Furthermore, both activities require stamina and physical fitness, and for this reason, dance is often classified as a form of exercise [6].

Exercise dependence has been described as a craving for leisure-time physical activity, resulting in uncontrollable excessive exercise behaviour [7–10]. Griffiths [11] suggested that—like most behavioural addictions—exercise addiction manifests in six general symptoms: salience, mood modification, tolerance, withdrawal symptoms, conflict and relapse [12]. Apart from the decline in mental health, exercise dependence and/or addiction is associated with body image distortions [7,13]. The implications of such findings are considerable given that body image disturbances mediate the development of eating disorders [14,15]. Confirming these assumptions, research suggests strong links between exercise addiction and eating disorders [16,17]. Furthermore, it appears that exercise dependence where running is the primary activity is associated with a rigid and inflexible personality pattern [18]. These data suggest that excessive exercising is linked to psychopathology and is a potentially self-harming behaviour.

There are data from several empirical studies suggesting that psychopathology may be present in dance addiction similar to that in exercise addiction. For example, Pierce and Daleng [19] conducted a study with 10 elite ballet dancers and found that dancers rated thinner bodies as ideal and significantly more desirable than their actual body image despite being in the ‘ideal’ BMI range. Furthermore, dancers often continue to dance despite discomfort, “because of the embedded subculture in dance that embraces injury, pain, and tolerance” (p. 105) [20]. In a recent study, Targhetta et al. [21] assessed addiction to the Argentine tango. They found that almost half (45%) of the participants met the DSM-IV criteria of abuse, although a substantially lower prevalence rate (7%) was found when Goodman’s [22] more conservative criteria and higher threshold for addiction were taken into account. Nevertheless, the mental health of ‘addicts’ and ‘non-addicts’ was not assessed and compared in this study, nor has it been investigated by other previous studies. Furthermore, the empirical investigation of motivation underlying excessive dancing is also lacking in the field.

The present study proposes that excessive social dancing is associated with detriments to mental health. More specifically, the study aimed (i) to identify subgroups of dancers regarding addiction tendencies and (ii) to explore which factors account for the elevated risk of dance addiction. In the latter of these aims, demographic variables, (increased) dance activity, psychiatric distress, (decreased) wellbeing, borderline personality disorder symptoms, body shape dissatisfaction and eating disorder symptoms were expected to be contributing factors of dance addiction. Finally, the study aimed (iii) to explore the motivations underlying excessive dancing.

## Methods

### Participants and procedure

The study aimed to recruit individuals who danced salsa, Latin or ballroom for recreational purposes at least once a week. These three genres were selected because they are currently the most popular recreational form of dancing in Hungary. Data collection occurred online. The questionnaire (titled as assessing “the psychology of dance”) was posted on the most popular Latin dance website in Hungary (i.e., latinfo.hu) and was shared on *Facebook* during July and August 2013. Participation was voluntary and no incentives were given for participation. A total of 688 questionnaires were started but not all participants completed the whole survey. This left a total of 457 completed questionnaires. A further 10 questionnaires were excluded because these respondents indicated never to have danced the listed genres (i.e., salsa or ballroom) before. This resulted in 447 completed responses for data analysis. Informed consent was obtained from all participants via an online system. After introducing the goals of the study in detail, the participants were asked to tick into a box if they agreed to continue and participate in the study. The study protocol was approved by the Institutional Review Board of the Eötvös Loránd University.

### Measures

Major socio-demographic characteristics of the dancers (gender, age, education) were assessed. Intensity was operationalized as the number of hours spent in training and/or in a formal dance event in an average week. Experience was defined as the total number of active years spent in dance.

#### Dance Addiction Inventory (DAI)

The short, theory-based Exercise Addiction Inventory (EAI) [12,23] was adapted for dancing by exchanging the word “exercise” to “dance” (S1 Inventory). Furthermore, in order to reflect the change of the definition of addiction in DSM-5, the seventh criteria of ‘craving’ was added to the existing six items of the EAI. The Dance Addiction Inventory (DAI) contained seven items, rated 1 to 5 (where 1 = strongly disagree; 5 = strongly agree). Cronbach’s alpha for the whole scale was 0.78.

#### Brief Symptom Inventory (BSI)

The Brief Symptom Inventory [24] identifies self-reported clinically relevant psychological symptoms among individuals during the past seven days. Items are rated from 1 (not at all) to 5 (very much). The BSI consists of 53 items covering nine symptom dimensions: Somatisation, Obsession-Compulsion, Interpersonal Sensitivity, Depression, Anxiety, Hostility, Phobic Anxiety, Paranoid Ideation and Psychoticism. The Global Severity Index (GSI) is the sum total of all items and is referred to as psychiatric distress.

#### Mental Health Continuum—short form (MHC)

Subjective wellbeing was assessed by the Mental Health Continuum [25] which defines mental health as a syndrome of symptoms of positive feelings and positive functioning in life. Via self-administered questionnaire, respondents indicate how much of the time during the past 30 days they felt the symptoms of positive emotional, psychological and social affect on a scale of 1 (‘never’) to 6 (‘every day’) by means of 14 items. On the present sample the internal consistency (Cronbach’s alpha) of the total scale was 0.95.

#### Body mass index and body image

Body Mass Index (BMI) was defined as the individual’s weight (in kilograms) divided by the square of their height in meters (kg/m^2^). Values between 18.50 and 24.99 are considered normal [26]. Body shape dissatisfaction (BSD) was calculated based on the Body Shape Test [27] by calculating the difference between ideal and actual body shape based on pre-determined pictures. The greater the difference, the more dissatisfied the person is with their body shape. Body weight discrepancy was defined as the difference between the individual’s ideal and actual weight.

#### Eating disorder

Eating disorder symptoms were identified using the SCOFF questionnaire [28,29]. The SCOFF is a brief tool designed to detect the core features of eating disorders (i.e., anorexia nervosa and bulimia nervosa) via five yes/no questions. Having two or more affirmative answers indicates the presence of an eating disorder.

#### Borderline personality traits

Borderline symptoms were assessed using the McLean Screening Instrument for Borderline Personality Disorder (MSI-BPD) [30]. The instrument is based on the DSM-IV criteria of borderline personality disorder and is suitable for use as a severity index for borderline symptoms. The ten items are rated yes/no, and ≥ 7 affirmative answers indicate the probable presence of borderline personality disorder. The MSI-BPD had acceptable sensitivity (81%) and specificity (85%) when the BPD module of the Diagnostic Interview for DSM-IV Personality Disorders [31] was applied as external criterion. The scale also had acceptable reliability (0.70) in the present sample.

#### Dance motives

To assess the motives for dance, the Dance Motivation Inventory (DMI) [32] was administered to all dancers. The DMI was developed based on the experiences of dancers and additional items from exercise addiction literature. Items are rated from 1 (strongly disagree) to 5 (strongly agree). In total, the 29 items loaded on eight factors with good reliability in the present sample: Fitness (0.91), Mood Enhancement (0.81), Intimacy (0.80), Socialising (0.85), Trance (0.90), Mastery (0.73), Self-confidence (0.81) and Escapism (0.79).

### Statistical analysis

Two types of analyses were applied: a person-centred approach (latent profile analysis) and various other tests using continuous variables (such as factor analysis and regression). The reason for applying both approaches was two-fold. First the study aimed to identify subgroups of dancers according to their addiction profile, thus subgrouping via latent profile analysis was the most suitable method of calculation. The other aim of this study was to explore which variables have predictive value on dance addiction. A variable-centred approach was considered most suitable to investigate this aim.

In order to identify the groups of users with high risk of problematic use of dancing, cross-sectional mixture models were estimated with the seven items of DAI as class indicators. A special case of mixture models is latent profile analysis (LPA) [33,34] which is a method for identifying unmeasured class membership among individuals using continuous observed variables (in this case the factor scores of DAI). LPA was performed with two to six classes on the full sample of dancers (N = 447). In confirmatory factor analysis (CFA) mixture modeling the factor mean varies across classes while all other model parameters are held equal [35]. CFA mixture modeling was carried out using maximum likelihood estimation.

In the process of determining the number of latent classes, the Bayesian information criteria (BIC) parsimony index was used, alongside with the Akaike Information Criteria (AIC), sample size adjusted BIC (SSABIC) and entropy. Lower BIC, AIC and SSABIC values indicate better fit of the model and higher entropy indicates better classification quality. Clark and Muthén [36] suggest 0.4, 0.6 and 0.8 as representing low, medium, and high entropy, respectively. In the final determination of the number of classes, the likelihood-ratio difference test (Lo-Mendell-Rubin Adjusted LRT Test, LMR) was also used. This compares the estimated model with a model having one less class than the estimated model [37]. A low *p* value (<.05) indicates that the model with one less class is rejected in favour of the estimated model.

Following the establishment of the number of latent classes, Wald tests were carried out to test the validity of the latent classes by comparing the classes along a number of variables (i.e., demographic variables, dance activity, general wellbeing, eating disorders and borderline symptoms) relevant to dance addiction. For these comparisons, Wald’s chi-square test of mean equality for latent class predictors in mixture modeling was used (for description of analysis, see www.statmodel.com/download/meantest2.pdf). The greater the value of Wald chi-square, the larger the group differences on the given variable. However, instead of using class membership as exact observed variables, class membership probabilities as auxiliary variables (covariates) were applied. The advantage of the probability approach is that it takes the posterior probability of class membership into account by using an individual’s logit-transformed posterior class probability as the outcome [36]. This means that instead of categorically belonging to one class or another, an item can have a specific probability of belonging (ranging between 0 and 1). This method therefore provides a more precise (i.e., weighted) estimate of class membership in the analyses than the traditional (i.e., non-weighted) method of classification.

To assess associations and group differences, a series of Pearson product-moment correlations (i.e., between DAI and continuous variables), independent sample t-tests (i.e., between DAI and bivariate variables) and ANOVAs (i.e., between DAI and ordinal variables) were performed. All continuous variables were normally distributed according to the Q-Q plots.

To predict DAI scores, multiple linear regression model was applied [38]. Block 1 contained the variable (Intensity) which is expected to predict DAI using Enter method. Block 2 (GSI, BPD and SCOFF) and Block 3 (each DMI factor) contained exploratory variables using Stepwise method of entry. In order to perform a linear regression on a continuous variable, multicollinearity was verified. The Variance Inflation Factor (VIF) is based on the proportion of variance the independent variable shares with the other independent variables in the model. As a rule of thumb, a VIF value greater than 4 would indicate inflated standard errors of regression coefficients [39]. In the present sample, VIF values remained well below threshold, ranging between 1.00 (Model 1 Intensity) and 1.25 (Model 5 DMI Escapism). Data analysis was carried out with MPlus 6.0 [35] (CFA mixture modeling, LPA and Wald test) and with SPSS 17.0 for Windows (the remainder).

## Results

### Characteristics of the sample

Mean age of the participants was 32.8 years (SD = 8.6) and approximately two-thirds of the sample was female (68%, n = 305). The majority of the sample (n = 311, 70%) had graduate education, 28% (n = 126) had secondary school education and the remainder (2%, n = 9) reported having an education lower than secondary school. Regarding dance intensity, the majority (57%) danced one to four times a week (at lesson and/or at formal dance events), 7% danced less and 36% more than this. In relation to dance experience, 9% of the sample danced for less than a year, 26% danced for 1 or 2 years, 29% danced for 2 to 4 years, and 36% danced for more than four years. Mean BMI was 22.38 (SD: 3.45).

### Latent profile analysis and factor mixture modelling

A latent profile analysis was performed on the items of DAI to identify risk groups of dance addiction. Two to six class solutions were estimated. Table 1 demonstrates that the AIC, BIC and sample-size adjusted BIC continued to decrease as more latent classes were added. Based on AIC, BIC and SSABIC, the four-latent-class solution was noted. After inspection of entropy, the four-class solution reached the maximum level, but the five-class solution also provided an adequate level of entropy (0.84). Based on the LMR test, the five-class solution was accepted.

**Table 1:** Fit indices for the latent class and CFA factor mixture modeling.

Models	AIC	BIC	SSABIC	Entropy	LMR test	*p*
*Latent class analyses*						
2 classes	9620	9710	9641	0.76	544.9	<0.001
3 classes	9410	9537	9442	0.85	217.5	<0.001
4 classes	9348	9504	9384	0.87	89.6	0.007
**5 classes**	**9259**	**9448**	**9302**	**0.84**	**94.0**	**0.044**
6 classes	9218	9439	9268	0.84	9.6	0.241
*CFA factor mixture modeling*						
2 classes	9485.5	9579.9	9506.9	0.42	**-**	**-**
**3 classes**	**9485.9**	**9588.5**	**9509.2**	**0.56**	**-**	**-**
4 classes	9499.4	9610.2	9524.5	1.00	**-**	**-**
5 classes	9493.9	9612.9	9520.8	0.70	**-**	**-**
6 classes	9501.5	9628.7	9530.4	0.78	**-**	**-**

Note: CFA = Confirmatory Factor Analysis; AIC = Akaike Information Criteria; BIC = Bayesian Information Criteria; SSABIC = Sample size adjusted BIC. LMR test = Lo-Mendell-Rubin adjusted likelihood ratio test value; p = p-value associated with LMR test.

Lower BIC, AIC, SSABIC and LMR values indicate better fit of the model and higher entropy indicates better classification quality. The most appropriate class solution is in bold. Based on the fit indices, latent classes were formed based on the results of the latent class analysis rather than CFA factor mixture modeling.

In addition to LPA, CFA mixture modeling was also performed with the same class indicators (the seven items of DAI). Table 1 presents the fit indices and entropy. The two-class solution yielded the lowest AIC, BIC and SSABIC values. However, entropy was unacceptably low (0.42). Therefore, the three-class solution was opted for where AIC and BIC values were the second-lowest, and SSABIC values and entropy (0.56) were also acceptable. Nevertheless, when comparing the five-class LPA solution and the three-class CFA mixture modeling solution, both the fit indices (AIC, BIC and SSABIC) and entropy was better for the five-class solution. Therefore the three-class solution of the CFA mixture model was rejected in favour of the five-class solution obtained via LPA.

**Fig 1:**
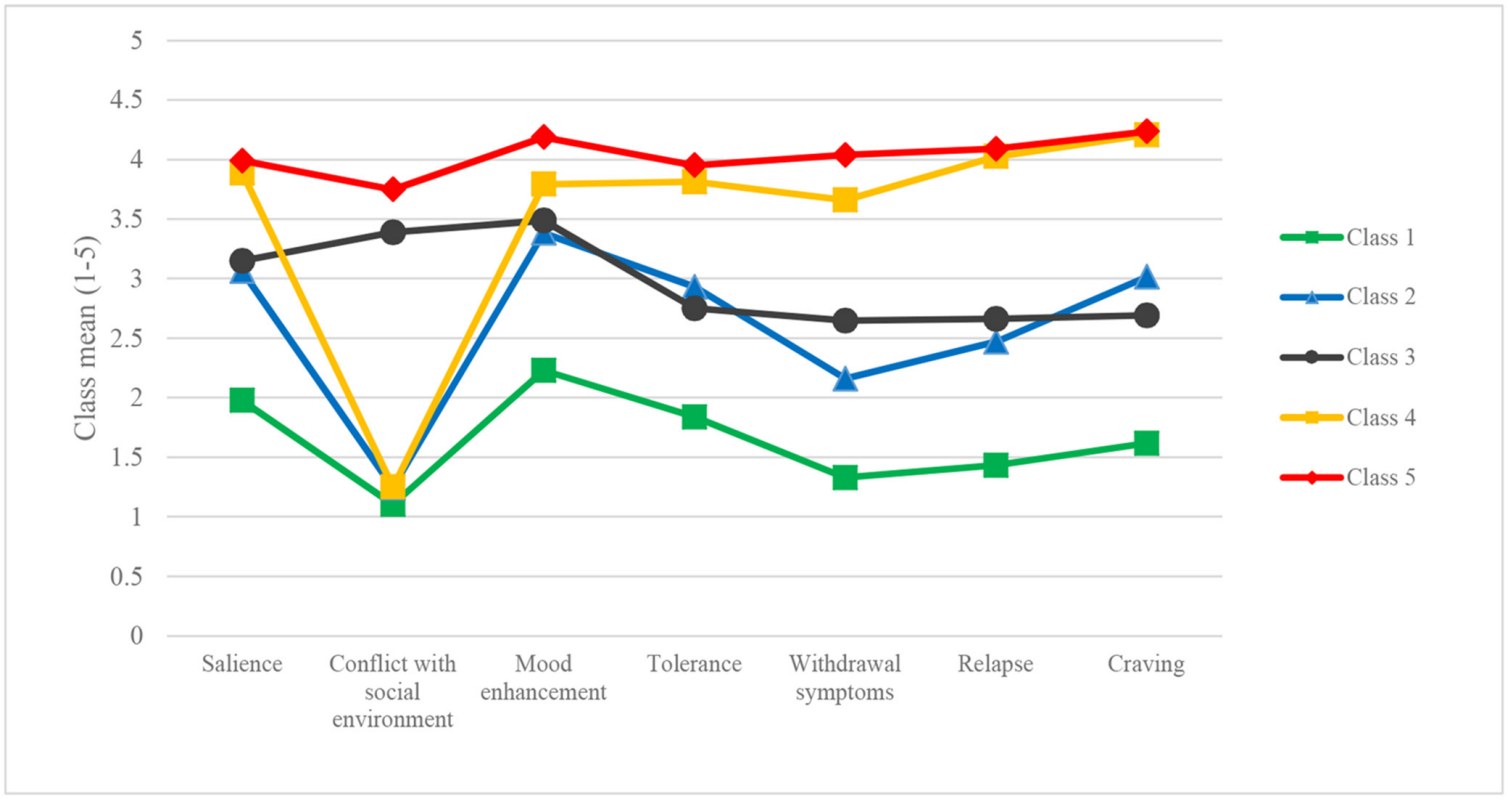
Dance Addiction Inventory item means per DAI class.

The features of each class are presented in Fig 1. The first class (low-risk) represented those dancers that scored below the average on all dimensions of problematic behaviour. The second class (medium-risk without social conflicts) and the third class (medium-risk with social conflicts) of dancers represented mild problematic behaviour. The fourth class (at-risk with no social conflict) was close to problematic behaviour, although participants reported no conflict with the social environment. Finally, the fifth class (at-risk with social conflict) represented a high risk of problematic behaviour. In this latter group, all factors showed an elevated level of all problematic dimensions and encompassed 11.4% of the sample. Group differences between classes on DAI were all significant (see Table 2).

**Table 2:** Latent class differences regarding demographic variables, mental health, body image and eating disorder symptoms.

	Association with DAI total score	Class 1, n = 40	Class 2, n = 56	Class 3, n = 189	Class 4, n = 111	Class 5, n = 51	Wald χ^2^
**Gender (%women)**	t = -1.03	64%	69%	56%	72%	71%	3.77
**Age**	r = -0.12*	33.5^4^	33.6^4^	34.3^4^	30.7^1,2,3^	32.5	6.25
**Education** ^**¥**^	F = 3.71**	3.8 (0.1)^4^	3.72 (0.1)^4^	3.74 (0.1)^4^	3.48 (0.1) ^1,2,3^	3.7 (0.1)	12.51*
**Intensity (hours per week)**	r = 0.27***	3.8 (0.2)^3,4,5^	4.3 (0.1)^1,5^	4.4 (0.3)	4.5 (0.1)^1^	5.0 (0.3)^1,2^	14.00**
**Experience (years of active dance)**	r = 0.06	5.6 (0.4)	5.6 (0.2)	6.2 (0.4)	6.0 (0.3)	5.9 (0.4)	2.6
**Global Severity Index**	r = 0.18***	76.0 (3.4)^4,5^	80.4^5^ (2.0)	81.4 (3.7)	84.9 (2.5)^1^	89.1^1,3^ (3.3)	7.73^+^
**Mental Health Continuum**	r = -0.02	56.4 (1.8)	56.2 (0.9)	55.2 (1.9)	56.5 (1.2)	54.4 (1.8)	1.25
**BPD symptoms**	r = 0.18***	1.97 (0.3)^4,5^	2.35 (0.2)^4^	2.60 (0.4)	3.15 (0.2)^1,2^	2.86 (0.3)^1^	10.50*
**Presence of BPD**	t = -0.92	4 (10%)	4 (7.1%)	7 (3.7%)	8 (7.2%)	2 (3.9%)	
**BMI**	r = 0.02	22.1 (0.5)	22.6 (0.3)	22.9 (0.5)	22.0 (0.3)	22.4 (0.5)	2.32
**Body Shape Dissatisfaction**	r = 0.12*	0.30 (0.2)^3^	0.61 (0.1)	0.85 (0.2)^1^	0.64 (0.12)	0.74 (0.2)	6.50^+^
**Weight discrepancy**	r = 0.09*	1.87 (0.8)	2.84 (0.5)	2.97 (0.67)	2.47 (0.56)	3.54 (0.9)	2.7
**SCOFF (eating disorder symptoms)**	r = 0.19***	0.25 (0.1)^5^	0.31 (0.1)^5^	0.37 (0.1)^5^	0.43 (0.1)	0.66 (0.1)^1,2,3^	7.81^+^
**Presence of an eating disorder**	t = 1.42	1 (2.5%)	4 (7.1%)	13 (6.9%)	9 (8.1%)	7 (13.7%)	
**Dance Addiction Inventory**	-	11.5 (0.4)^2,3,4,5^	18.3 (0.2)^1,3,4,5^	20.8 (0.5)^1,2,4,5^	24.6 (0.3)^1,2,3,5^	28.3 (0.4)^1,2,3,4^	1052.1***

Note: The Class cells (1–5) contain the mean and standard deviation (SD) of the corresponding variable in the row.

Superscript numbers (^1, 2, 3, 4, 5^) reflect significant (p < 0.05) difference between the mean of the given class (indexed cell) and the mean of the class given in the index—within the same variable (row)—according to Wald χ^2^ test of mean equity for latent class predictors; r = correlation coefficient, t = independent sample t-test value, F = ANOVA value.

^+^p<0.1

*p<0.05

**p<0.01

***p<0.001

BPD = Borderline personality disorder, BMI = Body Mass Index.

^¥^Education was coded as university (= 4), high school (= 3), vocational (= 2) and lower than 12 classes (= 1)

Differences between the latent classes on the given addiction dimensions were mostly significant (*p*<0.05) (Salience: Class 1 vs. 2, 4, 5; Class 2 vs. 3, 4, 5; Class 3 vs. 4, 5, Conflicts with social environment: Class 1 vs. 2, 3, 4, 5; Class 2 vs. 5; Class 3 vs. 5, Class 4 vs. 5, Mood enhancement: Class 1 vs. 2, 5; Class 2 vs. 3, 4, 5; Class 3 vs. 4, 5, Tolerance: Class 1 vs. 2, 4, 5; Class 2 vs. 3, 4, 5; Class 3 vs. 4, 5, Withdrawal symptoms: Class 1 vs. 2, 3, 4, 5; Class 2 vs. 3, 4, 5; Class 3 vs. 4, 5, Relapse: Class 1 vs. 2, 4, 5; Class 2 vs. 3, 4, 5; Class 3 vs. 4, 5 and Craving: Class 1 vs. 2, 4, 5; Class 2 vs. 3, 4, 5; Class 3 vs. 4, 5.

### Class differences

**Fig 2:**
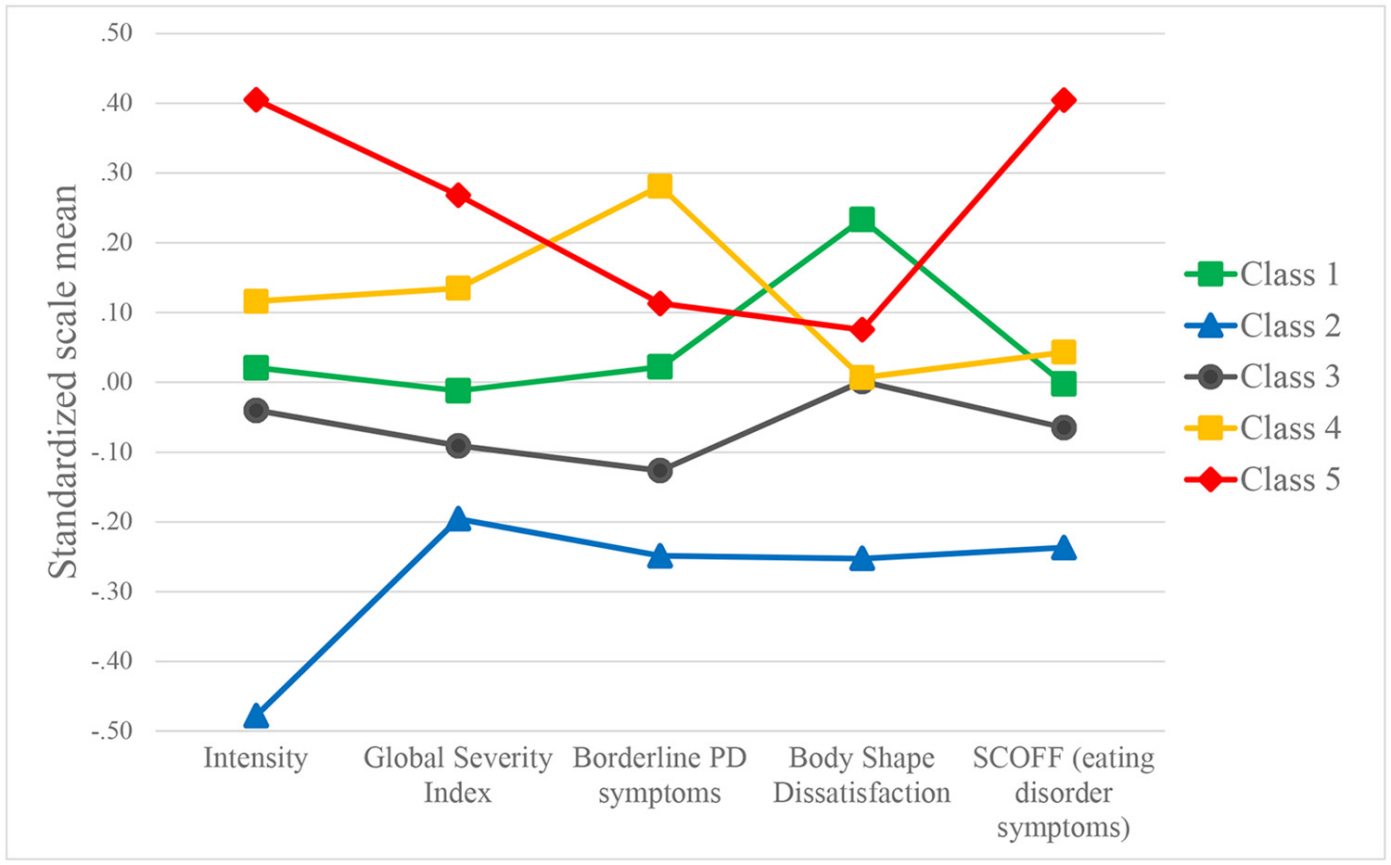
Latent class means across variables.

Table 2 depicts group differences on the measured variables between latent classes. Class 5 has high values on Intensity and Global Severity Index (both compared to Class 1 and 2), MSI-BPD (compared to Class 1), and SCOFF (compared to Classes 1, 2 and 3), but they are similar to the other groups in terms of demographic variables and BMI. The other problematic group (Class 4) has increased values on Intensity and GSI (both compared to Class 1) and MSI-BPD (compared to Classes 1 and 2). Latent class differences (where Wald χ^2^ *p*<0.1) are depicted on Fig 2.

### Regression models

In order to account for possible overlap among variables, regression using the stepwise method was conducted. Adding variables in three separate blocks (Block1: Intensity, Block 2: SCOFF, GSI, BPD, Block 3: DMI factors) resulted in five separate models with DAI scores as the dependent variable (see Table 3). Each model has significantly better predictive potential than the previous one. Intensity was a constant predictor of DAI variance. The predictive power of BPD decreased across the models as more variables were added. Although GSI was entered, it did not have any predictive effect on DAI in any of the models. The final model (Model 5) incorporated DMI-Escapism, Intensity, DMI Mood Enhancement, SCOFF and BPD as predictor variables (in decreasing effect) and explained 41% of DAI total variance.

**Table 3:** Regression model coefficients.

	Unstandardized		Standardized	95% Confidence Interval	
	ß	Std. Error	*ß*	Lower Bound	Upper Bound
*Model 1*					
**(Constant)**	15.80	0.80		14.23	17.36
**Intensity**	1.03	0.17	0.27	0.69	1.36
*Model 2* (R^2^ = 0.12)					
**(Constant)**	13.95	0.86		12.26	15.64
**Intensity**	1.11	0.17	0.30	0.78	1.44
**BPD**	0.58	0.12	0.22	0.35	0.81
*Model 3* (R^2^ = 0.14)					
**(Constant)**	13.93	0.85		12.25	15.60
**Intensity**	1.08	0.17	0.29	0.75	1.41
**BPD**	0.48	0.12	0.19	0.25	0.72
**SCOFF**	1.04	0.39	0.12	0.28	1.81
*Model 4* (R^2^ = 0.39)					
**(Constant)**	23.79	1.02		21.79	25.79
**Intensity**	0.86	0.14	0.23	0.59	1.14
**BPD**	0.15	0.10	0.06	-0.05	0.36
**SCOFF**	0.79	0.33	0.09	0.15	1.44
**DMI Escapism**	2.79	0.20	0.53	3.19	2.39
*Model 5* (R^2^ = 0.41)					
**(Constant)**	24.83	1.04		22.79	26.87
**Intensity**	0.83	0.14	0.22	0.55	1.10
**BPD**	0.21	0.10	0.08	0.01	0.41
**SCOFF**	0.89	0.32	0.11	0.26	1.53
**DMI Escapism**	2.49	0.22	0.47	2.91	2.06
**DMI Mood Enhancement**	1.34	0.35	0.15	2.03	0.66

Note: DAI total score was added as dependent variable. Each model is significantly better than the previous one (ΔF_M1_ = 35.80***, ΔF_M2_ = 24.84***, ΔF_M3_ = 7.19** ΔF_M4_ = 186.35*** ΔF_M5_ = 15.00***, where ***p<0.001, **p<0.01)

## Discussion

The purpose of the present study was to explore the effect of dance addiction on mental health and wellbeing. To the authors’ knowledge, this is the first study to explore the psychopathology and motivation behind dance addiction. Based on the criteria of addiction by Griffiths [11], five distinct latent classes were established. Classes 1, 2 and 3 encompassed low to moderate risk dancers. About one-quarter of the sample reported high values on all seven criteria of addiction but reported no conflict with the social environment (Class 4). Finally, 11% of dancers belong to the fifth (i.e., most problematic) class scoring high on all addiction symptoms. This is in line with previous research into muscle dysmorphia [40], although no previous study has explored the latent profile of problematic exercisers.

The results also indicated that dance addiction was associated with mild psychopathology, especially with elevated number of eating disorder symptoms, and to a lesser extent, borderline personality traits. Again, this result is in line with previous studies reporting elevated psychopathological factors behind problematic exercisers [6,15,41]. Escapism as a motivational factor (and to a lesser extent Mood Enhancement) is an especially strong indicator of dance addiction, even after accounting for the overlap with other possible mediators such as distress. The role of Escapism has already been much reported in other types of behavioural addiction such as gambling and video gaming [42–44]. Here, escapism as a motivational factor refers to dancing in order to avoid feeling empty or as a mechanism to deal with everyday problems. This provides evidence that to a minority of individuals, dance addiction may be a maladaptive coping mechanism. These results provide support for the notion that excessive dancing constitutes a potential addiction problem for a minority of individuals.

Although the fifth, problematic (high-risk) class is not a clinically diagnosed “addicted” group, it appears that the prevalence of high-risk problematic dancers (11%) is substantially lower than calculated from the DSM-IV criteria of abuse (45%) in a previous study examining tango dancers but slightly higher than the prevalence following Goodman’s criteria of addiction (7%) [21]. Furthermore, in the present study, external validating factors confirmed the existence of a problematic group of dancers. For example, in the problematic group (Class 5), the prevalence of eating disorders was about twice as great as in any other class. Moreover, given that SCOFF does not contain items of exercise as a purging behaviour, the prevalence of eating disorders among problematic dancers was probably underestimated in the current sample. Future studies should also assess whether eating disorder is primary or secondary to dance addiction, that is to say, whether the purpose of excessive dancing is weight-control and/or the motivation to perform leads to disturbances in eating patterns [45]. On the other hand, when mental health was approached with positively-worded items via the Mental Health Continuum, there were no group differences. This is due to the fact mental ‘healthiness’ (i.e., emotional, psychological, and social wellbeing) and mental illness (i.e., major depressive episode and addiction problems) constitute separate unipolar dimensions [46]. On the other hand, the participants comprised a highly educated sample, and high education is associated with lower happiness [47] that is not true for distress where high education is associated with fewer psychiatric symptoms [48]. Finally, it is also possible that the different classes captured different stages of developing addiction thus the differences between classes on the given variables.

Although distress correlates with dance addiction, this association disappeared when other measures were added to the regression model. This may indicate that distress is not directly associated with problematic dancing and that it may arise from other problematic factors such as having an eating disorder. One of the common elements between behavioural and chemical addiction is that they both involve the satisfying of short-term needs at the expense of longer-term negative effects [11,49,50]. From this perspective, excessive behaviours serve as a coping mechanism to regulate unpleasant feelings and cognitive affects [11]. Indeed, the fact that Escapism and Mood Modification above all other motivational factors were associated with dance addiction is in line with the theory of excessive behaviours as coping mechanisms against everyday distress.

This study is not without its limitations. The total population of dancers (and therefore the response rate and representativeness of the results) are unknown. Many individuals did not complete the questionnaire because it was either too long and/or questions may have been too personal and uncomforting. If the latter is true, then the number of dancers at risk of addiction was probably underestimated in the present sample. The data were also self-report and therefore subject to possible biases (e.g., social desirability bias, recall bias, etc.). Furthermore, and similarly to most other Internet-based recruitment methods, self-selection could also have led to possible bias.

Given the lack of research in the field, it is paramount to further clarify the concept of dance addiction. For example, it remains a question as to whether dance addiction is conceptually different from exercise addiction. Although the motivational factors are somewhat different from those underlying exercise dependence [32], the question to what extent do the two concepts overlap remains unanswered. An even more important task is to develop a new measure for dance addiction with a valid cut-off value. Such an instrument should pay close attention to the role of social conflicts as a result of excessive and/or problematic dancing. Given that dancing is a social activity, social conflicts may not arise when the person has only fellow dancers as partners or friends—therefore, the risky behaviour may remain somewhat hidden. Additional items should assess physical consequences of problematic dancing such as the number of injuries and the prevalence of dancing despite being injured. Items exploring negative consequences (such as tiredness at work) should also be added. Given the fact that “runners high” is a good predictor of compulsive running [18], it would be interesting to know whether a similar pattern is present in excessive dancing. Another question could examine the differences between amateur and professional dancers in terms of addiction tendency (although among professional dancers there may be a debate about whether their behaviour is dancing addiction or ‘workaholism’). Finally, it is not known whether the findings outlined here extend to other dance genres, therefore this could be the focus of future studies.

Dancing is very clearly a healthy activity for the majority of individuals therefore one should avoid overpathologizing the behavior [51]. However, excessive dancing may have problematic and/or harmful effects on the individual’s life just as any activity that has a rewarding value [52]. Although causality is yet to be established, clearly, dance addiction has the potential to be associated with mild psychopathology among a minority of individuals.

## Supporting Information

S1 Inventory (DOCX)

S1 Dataset (CSV)
