# Supplementary material for: An Empirical Investigation of Dance Addiction
Source: PLoS One. 2015 May 7;10(5):e0125988. doi: 10.1371/journal.pone.0125988 (PMC4423970; doi:10.1371/journal.pone.0125988)
Supplement: S1 Inventory — (DOCX) [file pone.0125988.s001.docx]

**S1 Inventory**

**Dance Addiction Inventory**

|  | **DSM-5 criteria of abuse** | **Exercise Addiction Inventory item** | **Dance Addiction Inventory** |
| --- | --- | --- | --- |
| 1. | **Salience** | Exercise is the most important thing in my life | Dance is the most important thing in my life |
| 2. | **Conflict with social environment** | Conflicts have arisen between me and my family and/or my partner about the amount of exercise I do | Conflicts have arisen between me and my family and/or my partner about the amount of dancing I do |
| 3. | **Mood enhancement** | I use exercise as a way of changing my mood | I use dancing as a way of changing my mood |
| 4. | **Tolerance** | Over time I have increased the amount of exercise I do in a day | Over time I have increased the amount of dancing I do in a day |
| 5. | **Withdrawal symptoms** | If I have to miss an exercise session I feel moody and irritable | If I have to miss a dance session I feel moody and irritable |
| 6. | **Relapse** | If I cut down the amount of exercise I do, and then start again, I always end up exercising as often as I did before | If I cut down the amount of dancing I do, and then start again, I always end up exercising as often as I did before |
| 7. | **Craving** | - | I feel a constant urge to dance |

*Note: Rating: 1 to 5, where 1=strongly disagree and 5=strongly agree*
